# Prevalence and Possible Factors of Myopia in Norwegian Adolescents

**DOI:** 10.1038/s41598-018-31790-y

**Published:** 2018-09-07

**Authors:** Lene A. Hagen, Jon V. B. Gjelle, Solveig Arnegard, Hilde R. Pedersen, Stuart J. Gilson, Rigmor C. Baraas

**Affiliations:** National Centre for Optics, Vision and Eye Care, Faculty of Health and Social Sciences, University of South-Eastern Norway, Kongsberg, Norway

## Abstract

East Asia has experienced an excessive increase in myopia in the past decades with more than 80% of the younger generation now affected. Environmental and genetic factors are both assumed to contribute in the development of refractive errors, but the etiology is unknown. The environmental factor argued to be of greatest importance in preventing myopia is high levels of daylight exposure. If true, myopia prevalence would be higher in adolescents living in high latitude countries with fewer daylight hours in the autumn-winter. We examined the prevalence of refractive errors in a representative sample of 16–19-year-old Norwegian Caucasians (n = 393, 41.2% males) in a representative region of Norway (60° latitude North). At this latitude, autumn-winter is 50 days longer than summer. Using gold-standard methods of cycloplegic autorefraction and ocular biometry, the overall prevalence of myopia [spherical equivalent refraction (SER) ≤−0.50 D] was 13%, considerably lower than in East Asians. Hyperopia (SER ≥ + 0.50 D), astigmatism (≥1.00 DC) and anisometropia (≥1.00 D) were found in 57%, 9% and 4%. Norwegian adolescents seem to defy the world-wide trend of increasing myopia. This suggests that there is a need to explore why daylight exposure during a relatively short summer outweighs that of the longer autumn-winter.

## Introduction

East and Southeast Asia have experienced an excessive increase in myopia in the past few decades, with more than 80% of the younger generation now affected^1,2^. Myopia is a major health concern^3–5^, as myopia, and in particular high myopia, may lead to potentially sight-threatening secondary ocular pathology^6^. The “epidemic” scale of myopia is most commonly observed in highly economically developed countries, where children complete secondary education and many undertake upper- and post-secondary studies, combined with limited time spent outdoors^7,8^.

Environmental and genetic factors are both assumed to contribute in the development of refractive errors^9,10^, although there is no general agreement on the etiology of myopia. The environmental factor argued to be of greatest importance in preventing myopia is time spent outdoors prior to myopia *onset*^11–13^ (it is debated whether time outdoors has an effect on myopia *progression*^14–19^). A dose-response relationship between daylight (outdoor) exposure and ocular axial elongation (associated with developing myopia) has been inferred^17^. Reported seasonal variation in axial length growth and myopia progression (with decreased eye growth and decreased myopia progression in periods with increased number of daylight hours^20,21^) is often cited in support of the protective effect of outdoors. Such an explanation warrants further examination and calls for refractive error data from different parts of the world^3,22^, in particular countries with high performing education systems and differing levels of seasonal variation in daylight.

**Figure 1:**
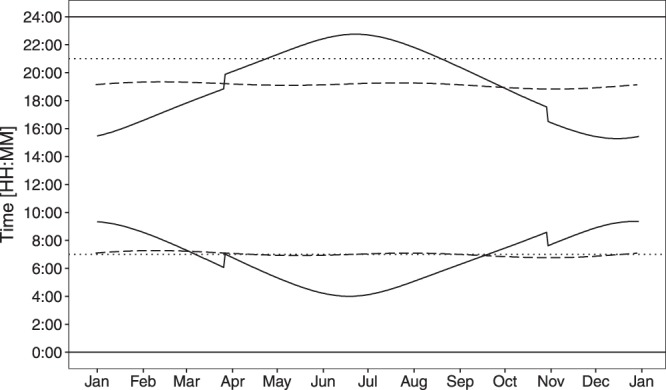
Seasonal variation in sunrise and sunset time. The solid line shows the seasonal variation in sunrise and sunset time in Southeast Norway (60° North, 9° East; range of daylight hours: 5 h 59 min – 18 h 44 min). The sudden change in late March and October is due to daylight saving time. For a comparison, the dashed line shows the sunrise and sunset time in Singapore (1° North, 103° East; range of daylight hours: 12 h 3 min – 12 h 12 min)^23^. The dotted lines show the amount of daylight available for a child sleeping 10 hours each night.

Norway’s northern latitude stretches from 58° to 71° North, with even those living in Southeast Norway (60° North) experiencing large seasonal variation in daylight exposure, from less than 6 hours in December to around 19 hours in June (Fig. 1)^23^. Norway is a highly economically developed country, ranked as number 1 in the Human Development report 2016, with high gender equality^24^. Norwegian children start primary school at age 6 years and complete 10 years of compulsory schooling before reaching upper secondary school, at age 16 years. Most of today’s adolescents will also have attended kindergartens from age 1–5 years (76.2% in 2005)^25^. The Norwegian education system is high-performing, as classified by the Organisation for Economic Co-operation and Development (OECD) Programme for International Student Assessment (PISA), with both mean performance and the proportion of top performers above the OECD average in science, reading and mathematics^26^. Near work includes high usage of near electronic devices (NED) at school and at home, with the use of NED reported to be above the OECD average^27^.

If high levels of daylight exposure are necessary to protect against myopia, it is reasonable to hypothesize that myopia onset will occur earlier, progression will be faster, and prevalence will be higher in adolescents living in countries with relatively few daylight hours across an extended (5–6 months of autumn-winter) period^28^, particularly so, if combined with a high level of near work^29,30^. The current study tested this hypothesis. Its aim, therefore, was to examine the prevalence of refractive errors in adolescents in Southeast Norway and assess the relationship between refractive errors, ocular biometry, sex and environmental factors such as self-reported time spent on activities outdoors and indoors.

## Methods

### Study Population and Recruitment

A cross-sectional study was carried out on students from the only two upper secondary schools within a catchment area comprising five municipalities in Southeast Norway during 2015–2016. The catchment area is representative of the Norway population in terms of socio-demographic status (details are given in Supplementary Tables S1–S4), with 70.7% living in urban settlements and an average population densities of 4–36 persons/km^2^ ^31^. The total population of the region was 49,293 in 2016, with 1,737 of these aged 16–19 years^32,33^. The total student population of the two schools was 1,970 (age 16–24 years), 676 and 1,294 in the first and second schools respectively. The students attend school 5 days a week for 5–8 hours per day, with the school day beginning no earlier than 8 am; in addition, students undertake homework in the evenings and on weekends. By agreement with school administrators, we were given access to 898 students (45.6%) who were all invited to participate; all students in all three years in the first school and those in their first year (typical age 16–17 years) in the second school. The sample was representative of the school’s catchment area with respect to ethnicity and grade point averages (see Supplementary Tables S2 and S5). The study was carried out at the schools during normal school hours.

Verbal and written information about the study was given, and possible consequences of the study were explained to all participants before written informed consent was obtained. The research was approved by the Regional Committee for Medical Research Ethics for the Southern Norway Regional Health Authority and carried out in accordance with the principles embodied in the Declaration of Helsinki. A person aged 16 years or older is considered an adult and fully competent to consent to participate in research according to the Norwegian Health Research Act.

### Participants

Of those invited, a sample of 439 (48.9%) students aged 16–19 years [mean age (*SD*): 16.7 (±0.9) years, 41.9% males] agreed to participate in the study. Self-reported ethnicity was mainly European Caucasians (90.9%); other ethnicities were Asian (5.5%), African (1.4%), South American (0.9%), or mixed (defined as having parents of two different ethnicities, 1.4%).

Analysis beyond calculation of prevalence of hyperopia and myopia was limited to the participants who reported to have both grown up in Norway and who were of Northern European (Caucasian) ethnicity [n = 393, mean age 16.7 (±0.9) years, 41.2% males], hereafter termed Norwegians. This group included participants born in Norway (98.7%) and five participants born in a different Northern European country (1.3%; born in Denmark, Iceland, Germany and Holland), all of whom reported to have moved to Norway during their childhood. Removal of these five participants from the group had no overall effect on the results. The Norwegian participants were grouped according to sex and age for the purpose of analysis (16-years-olds: n = 224, 42.4% males; 17–19-years-olds: n = 169, 39.6% males).

### Cycloplegic Autorefraction and Other Measurements

Cycloplegic autorefractions were obtained with a Huvitz HRK-8000A Auto-REF Keratometer (Huvitz Co. Ltd., Gyeonggi-do, Korea), 15–20 minutes after instillation of topical 1% cyclopentolate hydrochloride (Minims single dose; Bausch & Lomb UK Ltd, England). One drop of cyclopentolate was used for blue- and green-eyed participants, and two drops for brown-eyed participants. The mean of five measurements automatically performed by the instrument (Huvitz HRK-8000A) were used for further analyses. One qualified optometrist (author JVBG) performed all autorefraction and biometry measurements.

Ocular axial lengths (AL) and corneal radii (CR) were measured with Zeiss IOLMaster (Carl Zeiss Meditec AG, Jena, Germany). Body height was measured with the Seca 217 stable stadiometer for mobile height measurement (Seca Deutschland, Hamburg, Germany).

### Questionnaire

Participants completed an online questionnaire, an adapted version of the one used in the Sydney Myopia study^34^, to obtain demographic data and to quantify the amount of time spent on various indoor and outdoor activities. Demographic data included place of birth, number of years lived in Southeast Norway, house type and distance to school. Information about access to, and use of, near electronic devices (NED; smart phones, tablets, computers) was also collected.

The reported mean hours per day spent on outdoor- and indoor- activities were calculated for those participants who completed all questions related to time spent on various activities [68.4%, n = 269, 40.1% males, mean age 16.7 (±0.9) years]. Indoor activities included mean time spent on reading and writing on paper (books, newspapers, magazines), use of NED, indoor sport (gymnastics, dance, ball games, etc) and other indoor activities (watching television, playing video games, hobbies, cooking, etc). Outdoor activities included mean time spent on outdoor sport (cycling, skiing, running, etc) and other outdoor activities (walking to school, hiking, fishing, hunting, spending time in the garden etc). The participants were asked to estimate the daily time usually spent on these activities for both weekdays and weekends and about what they do in the school’s recess time. They were given four categorical response options for the estimate of activity hours per day; “Not at all”, “Less than 1 hour”, “1–2 hours”, or “3 hours or more”. The mean numbers of activity hours per day were calculated using “0 hour”, “1 hour”, “2 hours” or “3 hours” for each option, respectively, as follows:

1 $$Mean\,hours\,per\,day=\,\frac{(hours\,spent\,on\,weekdays\times 5)+(hours\,spent\,on\,weekends\times 2)}{7}$$

Finally, the participants were asked to estimate the ratio of indoor to outdoor activities during their school holidays. Data were collected during February and March at both schools.

### Analysis

Spherical equivalent refractive errors (SER = sphere + ½ cylinder), specified in terms of a 13.5 mm vertex distance, were used to classify refractive errors. Myopia was defined as SER ≤ −0.50 D, emmetropia as −0.50 D < SER < + 0.50 D, and hyperopia as SER ≥ + 0.50 D. The most positive meridian of the autorefractor measurement was defined as the sphere, and the prevalence of refractive astigmatism is reported as negative cylinder refraction ≥1.00 DC. SER, sphere and refractive astigmatism were all well correlated between the right and left eyes (SER: Spearman rho (*ρ*) = 0.94; sphere: *ρ* = 0.92; refractive astigmatism: *ρ* = 0.59; all *p* < 0.001), and thus only data from the right eye are presented. A SER-difference ≥1.00 D between right and left eye was defined as anisometropia. CR data represent the mean of the corneal radii measured in the flattest and steepest meridians. AL/CR-ratios were also calculated.

The Clopper-Pearson interval method and the method of Sison and Glaz were used for calculation of 95% binomial and multinomial proportion confidence intervals (CI), respectively. QQ-plots, histograms and the Shapiro-Wilk test were used to assess the normality of the variables. Means (±*SD*) are reported, in addition to the median (50th percentile) for non-normal data. The chi-square test, Fisher’s exact test, and independent sample *t*-test were used to assess differences in prevalence and mean values between groups. Maximum likelihood estimate was used to fit a suitable distribution to the data for SER^35^.

Linear regression analyses were performed with SER, AL, AL/CR-ratio and cylinder as the dependent outcome variables. Multivariate logistic regression analyses were performed, with the presence of myopia as the dependent outcome variable. Likelihood ratio tests were performed to compare models. Odds ratios (*OR*) and 95% CI are presented, with the significance level set at 0.05. All statistical analyses were performed using R statistical software, version 3.4.0^36^ including the packages MASS^35^ and gmodels^37^.

## Results

### Refractive Errors

Table 1 shows an overview of the prevalence of refractive errors by age and sex, independent of ethnicity (a) and for those defined as Norwegians (b). The overall prevalence of hyperopia and myopia was 55.4% and 13.4%, respectively. All results are from here on related to those defined as Norwegians.

**Table 1:** Mean spherical equivalent error SER (standard deviation, *SD*) in diopters [D] and the prevalence of refractive error type (%) for the right eyes categorized by age and sex of (a) all 16–19-year-olds, independent of ethnicity (*n* = 439), and (b) 16–19-year-old Norwegians (*n* = 393).

	Age (years)	Group	*n*	Mean (*SD*) SER [D]	Myopia % (CI)	Emmetropia % (CI)	Hyperopia % (CI)
(a) ALL ETHNICITIES	16–19	All	439	+0.51 (1.29)	13.4 (8.7–18.3)	31.2 (26.4–36.1)	55.4 (50.6–60.2)
		Females	255	+0.39 (1.30)	16.9 (10.6–23.1)	27.5 (21.2–33.7)	55.7 (49.4–62.0)
		Males	184	+0.67 (1.25)	8.7 (1.6–16.4)	36.4 (29.3–44.1)	54.9 (47.8–62.6)
	16	All	246	+0.59 (1.23)	11.0 (4.9–17.5)	31.3 (25.2–37.8)	57.7 (51.6–64.3)
		Females	139	+0.50 (1.10)	14.4 (6.5–22.9)	25.9 (18.0–34.4)	59.7 (51.8–68.2)
		Males	107	+0.72 (1.37)	6.5 (0.0–16.3)	38.3 (29.0–48.1)	55.1 (45.8–64.9)
	17–19	All	193	+0.40 (1.35)	16.6 (9.3–23.9)	31.1 (23.8–38.5)	52.3 (45.1–59.7)
		Females	116	+0.26 (1.50)	19.8 (11.2–30.0)	29.3 (20.7–39.5)	50.9 (42.2–61.1)
		Males	77	+0.60 (1.06)	11.7 (1.3–23.8)	33.8 (23.4–45.8)	54.5 (44.2–66.6)
(b) NORWEGIANS	16–19	All	393	+0.55 (1.29)	12.7 (7.9–18.0)	30.5 (25.7–35.8)	56.7 (51.9–62.0)
		Females	231	+0.45 (1.27)	15.6 (9.1–22.2)	28.1 (21.6–34.8)	56.3 (49.8–62.9)
		Males	162	+0.70 (1.30)	8.6 (1.2–16.7)	34.0 (26.5–42.1)	57.4 (50.0–65.5)
	16	All	224	+0.63 (1.23)	10.3 (4.0–17.1)	30.8 (24.6–37.7)	58.9 (52.7–65.8)
		Females	129	+0.56 (1.05)	13.2 (5.4–22.2)	25.6 (17.8–34.6)	61.2 (53.5–70.3)
		Males	95	+0.74 (1.43)	6.3 (0.0–17.0)	37.9 (28.4–48.6)	55.8 (46.3–66.5)
	17–19	All	169	+0.44 (1.37)	16.0 (8.3–23.7)	30.2 (22.5–37.9)	53.8 (46.2–61.6)
		Females	102	+0.31 (1.50)	18.6 (8.8–28.9)	31.4 (21.6–41.7)	50.0 (40.2–60.3)
		Males	67	+0.65 (1.12)	11.9 (1.5–24.7)	28.4 (17.9–41.1)	59.7 (49.3–72.5)

Prevalence is given with 95% confidence intervals (CI). Myopia was defined as SER ≤ −0.50 D, emmetropia as −0.50 D < SER < + 0.50 D, and hyperopia as SER ≥ + 0.50 D.

**Figure 2:**
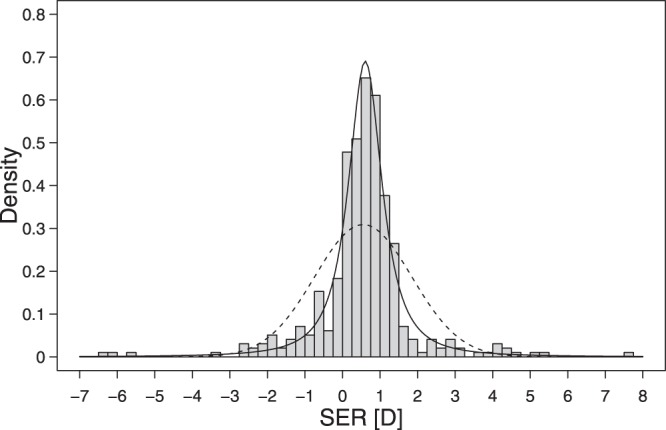
Distribution of SER. The leptokurtic distribution of cycloplegic SER [D] for the right eyes of 16–19-year-old Norwegians (*n* = 393; skewness = −0.24, kurtosis = 11.3). The dashed curve shows a normal distribution with the same mean and standard deviation as the data, and the solid curve shows a *t*-distribution fitted to the data by maximum likelihood [degrees of freedom (*df*) = 1.63, location (*m*) = 0.61, scale (*s*) = 0.50]^35^.

The prevalence of hyperopia and myopia in Norwegians was 56.7% and 12.7%, respectively. Figure 2 shows the leptokurtic distribution of SER [D] for 16–19-year-old Norwegians. The SER mean (±SD) was +0.55 (±1.29) D and median was +0.61 D (range: −6.45–7.71 D). Myopia was more prevalent among females than males [15.6% versus 8.6%, Fisher’s exact test, *p* = 0.046]. The prevalence of hyperopia decreased with age, with the prevalence of myopia increasing in parallel (Table 1b, column 6 and 8). However, the prevalence of high myopia, defined as SER ≤ −6.00 D, was very low, at 0.5% (CI: 0.1–1.8%). In contrast, the prevalence of moderate to high hyperopia, defined as SER ≥ + 2.00 D, was higher, at 6.4% (CI: 4.2–9.2%). Refractive astigmatism (≥1.00 DC) was found in 8.9% (CI: 6.3–12.2%) and anisometropia (≥1.00 D) in 3.6% (CI: 2.0–5.9%) of participants.

### Ocular Biometry and Body Height

Table 2 shows mean AL, CR and AL/CR categorized by age, sex, and refractive error. Mean AL was significantly longer (23.66 vs. 23.28 mm, *t*(391) = −4.46, *p* < 0.001) and mean corneal curvature (CR) was significantly flatter (7.87 vs. 7.78 mm, *t*(305) = −3.00, *p* = 0.003) in males compared with females. Overall, AL and CR were highly correlated (Pearson; *r* = 0.53 in females, *r* = 0.69 in males, *p* < 0.001), and both AL and AL/CR were significantly negatively correlated with SER in both males and females (AL: *r* = −0.62, (females), *r* = −0.47 (males), *p* < 0.001; AL/CR: *r* = −0.84 (females), *r* = −0.77 (males), *p* < 0.001).

**Table 2:** Mean (*SD*) axial length (AL), corneal radius (CR) and AL/CR-ratio for the right eye of 16–19-year-old Norwegians (*n* = 393) categorized by age, sex, and refractive error.

Age		*n*	SER [D] Mean (*SD*)	AL [mm] Mean (*SD*)	CR [mm] Mean (*SD*)	AL/CR Mean (*SD*)
16–19	All	393	+0.55 (1.29)	23.44 (0.86)	7.82 (0.27)	3.00 (0.09)
	Females	231	+0.45 (1.27)	23.28 (0.83)	7.78 (0.25)	2.99 (0.10)
	Males	162	+0.70 (1.30)	23.66 (0.86)	7.87 (0.30)	3.01 (0.09)
	Myopes	50	−1.60 (1.34)	24.22 (0.79)	7.74 (0.25)	3.13 (0.09)
	Emmetropes	120	+0.18 (0.23)	23.51 (0.75)	7.77 (0.27)	3.03 (0.07)
	Hyperopes	223	+1.23 (0.97)	23.22 (0.83)	7.86 (0.27)	2.95 (0.07)
16	All	224	+0.63 (1.23)	23.38 (0.82)	7.81 (0.28)	3.00 (0.09)
	Females	129	+0.56 (1.05)	23.21 (0.76)	7.78 (0.26)	2.99 (0.09)
	Males	95	+0.74 (1.43)	23.62 (0.85)	7.85 (0.31)	3.01 (0.10)
17–19	All	169	+0.44 (1.37)	23.51 (0.91)	7.83 (0.26)	3.00 (0.10)
	Females	102	+0.31 (1.50)	23.36 (0.91)	7.79 (0.24)	3.00 (0.11)
	Males	67	+0.65 (1.12)	23.73 (0.88)	7.89 (0.28)	3.01 (0.08)

The mean height of participants was 172.2 (±8.7) cm, with males being on average taller than females [179.2 (±7.1) cm vs. 167.3 (±6.0) cm, *t*(309) = 17.3, *p* < 0.001]. Height correlated with AL overall (Pearson; *r* = 0.28, *p* < 0.001) and in females (Pearson; *r* = 0.23, *p* < 0.001), but not in males (Pearson; *r* = 0.14, *p* = 0.08). Height did not correlate with SER.

### Outdoor and Indoor Activity Time

Times spent doing outdoor and indoor activities were calculated for the subset of Norwegian participants who answered all questions related to time spent on various activities. Although this subgroup represented only 68% of the total group, there were no differences between this smaller sample (*n* = 269) and the whole sample of Norwegian participants (*n* = 393) in prevalence of myopia (12.3% vs. 12.7%), emmetropia (30.9% vs. 30.5%) or hyperopia [56.9% vs. 56.9%; *χ*^2^(2) = 0.03, *p* = 0.984]. These participants reported to spend, on average, 3.8 (±1.8) and 10.5 (±2.4) hours per day outdoors and indoors, respectively. Most of the participants (93%) reported staying indoors in their school recess time. Myopes spent, on average, less time doing outdoor sport per day [0.93 (±0.8) h] than non-myopes [emmetropes and hyperopes combined: 1.32 (±1.0) h; *t*(267) = −2.24, *p* = 0.03], but total time spent outdoors was not associated with myopia [myopes: 3.65 (±1.5) h; non-myopes: 3.81 (±1.9) h; *t*(267) = 0.47, *p* = 0.64], neither was time spent on other activities. The hours spent on various indoor or outdoor activities also showed no significant correlations with either SER, astigmatism, AL or AL/CR-ratio.

Females and males spent, on average, the same amount of time outdoors [females: 3.71 (±1.7) h; males: 3.91 (±2.0) h] and indoors [females: 10.68 (±2.3) h; males: 10.26 (±2.4) h]. More than 97% of the students had both their own smart phone and laptop for use at school and for homework. The time spent using NED each day was the same for females and males [females: 5.01 (±1.5) h; males: 4.97 (±1.5) h].

Table 3 shows the models from the multivariate logistic regression, with myopia as the outcome variable, sex as potential confounder, and mean hours of different indoor and outdoor activities as the predictors (Model A). Likelihood ratio tests were used for manual backward selection (Model B). Model B confirmed a lack of significant association of myopia with indoor activities, but showed myopia to be associated with less time spent on outdoor sport (*OR* = 0.51, CI: 0.30–0.82, *p* = 0.007) and more time spent on other outdoor activities (*OR* = 1.49, CI: 1.04–2.15, *p* = 0.030), after adjustment for sex.

**Table 3:** Multivariate logistic regression models with myopia as the outcome variable. (Model A) mean hour of activity [h/day] as predictors and sex as a potential confounder.

	Model A			Model B		
	β	OR (95% CI)	*p*	β	OR (95% CI)	*p*
Intercept	−2.150	0.12 (0.02–0.75)	0.026	−2.041	0.13 (0.05–0.33)	<0.001
Sex, male	−0.625	0.54 (0.21–1.25)	0.164	−0.636	0.53 (0.21–1.21)	0.146
Sport outdoors	**−0.754**	**0.47** (**0.27–0.78)**	**0.005**	**−0.678**	**0.51** (**0.30–0.82)**	**0.007**
Other outdoors	**0.438**	**1.55** (**1.07–2.28)**	**0.022**	**0.400**	**1.49** (**1.04**–**2.15)**	**0.030**
Read paper	0.260	1.30 (0.75–2.23)	0.344			
NED	0.013	1.01 (0.78–1.31)	0.922			
Other indoors	−0.176	0.84 (0.59–1.18)	0.311			
Sport indoors	0.099	1.10 (0.72–1.72)	0.654			

AIC = 201.0. (Model B) mean hours of sport and other outdoor activities as the predictors, adjusted for sex. AIC = 195.1. Odds ratios (OR) and confidence intervals (CI) are presented.

Table 4 shows that 94% and 64% reported to spend half or more of the day outdoors in the summer and Easter holidays, respectively. More myopes (14%) than non-myopes (4%) reported to spend most of their time indoors during the summer holidays (Fisher’s exact test, *p* = 0.01), with no difference for the other holidays.

**Table 4:** Overview of duration, time and mean number of daylight hours for the school holidays in Norwegian upper secondary school, including proportion of students who reported to spend half of the day or more than half of the day outdoors in these periods.

	Duration of holiday (time of the year)	Mean # daylight hours in the period	Proportion (%)		Proportions (%) who spend most time indoors		
			Spend half of the day outdoors	Spend more than half of the day outdoors	Myopes	Non-myopes	*p*-value
Summer	8 weeks (mid June–mid August)	17 h 35 min	45	49	14	4	0.007*
Autumn	1 week (October)	11 h 5 min	38	9	47	53	0.447
Winter	1 week (February)	10 h 36 min	35	8	67	56	0.164
Spring (Easter)	1.4 weeks (March–April)	14 h 21 min	52	12	41	35	0.428

Proportions of the students who spend most time indoors are categorized as myopes and non-myopes (*p-*values were calculated using Fisher’s exact test for count data).

## Discussion

This is the first report on refractive errors in a representative sample of adolescents in Southeast Norway, with hyperopia found to be the most common type of refractive error. How does the refractive error profile of this adolescent population compare with other adolescent populations? The prevalence of moderate to high hyperopia (SER ≥ + 2.00 D) in this sample (6.4%) is higher than that reported for adolescents in both Asia (0.5–4.0%)^38–40^ and Australian European Caucasians (2.0%)^5^, but lower than among white adolescents in the UK (17.7%)^41^. Comparative data from other published studies on myopia prevalence are summarized in Table 5, with matched myopia definition. The prevalence of myopia is comparable with, albeit slightly lower than for Australian European Caucasians in Sydney^5^ and white adolescents in the UK^41^. It was lower than the 27.4% point estimate for myopia in the 15–19-year age group across Europe, calculated by random-effect meta-analysis and age-standardization by Williams *et al*.^42^ (mean SER for the two eyes ≤−0.75 D). The prevalence of myopia was also lower than that reported in a study of Swedish 12–13-year-olds^43^, though that study’s use of tropicamide 0.5% for accommodation control may have resulted in an artificially high myopia prevalence. The prevalence of myopia observed in the Southeast-Norwegian 16-year-olds is only slightly higher than that reported for 1-year-younger adolescents in rural Nepal, Iran and rural India^44–46^ (all considerably lower HDI than Norway). Noteworthy, the prevalence of myopia is considerably lower than that generally reported for adolescents in rural and urban parts of Asia^12,38–40,47–49^ [with comparable or lower human development index (HDI) than Norway]^24^, and Chile^50^ (considerably lower HDI than Norway). The ocular biometry data are consistent with the low myopia prevalence, with shorter axial lengths and lower average AL/CR than groups with higher myopia prevalence [cf. Table 2 with Lu *et al*.^51^ and Li *et al*.^52^].

**Table 5:** Summary of myopia prevalence (%) from this study (four leftmost columns) and from other studies (rows, bold), matched on myopia definition and best matched on age.

Age (years)	n	Myopia definition (SER)	Myopia prevalence (%) matched on myopia definition		Age (years)	n	Country	Ethnicity	HDI 2015^24^	Mean score in PISA 2015^82^	Average scale score TIMSS 2015^83^	Latitude
			Present study	Other studies					(HDI rank)	Science/Reading/Mathematics	Mathematics 8^th^ grade	
16	224	<0.00	17.4	**27.5**	**12–14**	**102**	**Norway** ^53^	**Not given**	0.949 (1)	498/513/502	512	60.4° N
		<−1.00	5.8	**13.7**	**12–14**	**102**	**Norway** ^53^	**Not given**	0.949 (1)	498/513/502	512	60.4° N
		≤−0.50	10.3	**44.9**	**12–13**	**1045**	**Sweden** ^43^	**Not given**	0.913 (14)	493/500/494	501	57.7° N
		<−0.50	10.3	**52.1**	**13–16**	**2069**	**Rural China** ^47^	**Han, Dai, Yi, Bai and other**	0.738 (90)	518/494/531	N/A	24.5° N
		≤−0.50	10.3	**38.8**	**14–15**	**905**	**Rural China** ^38^	**Not given**	0.738 (90)	518/494/531	N/A	40.1° N
		≤−0.50	10.3	**16.7**	**15**	**395**	**Suburban Chile** ^50^	**Not given**	0.847 (38)	447/459/423	427	33.5° S
		≤−0.50	10.3	**0.79**	**15**	**386**	**Rural Nepal** ^44, 84^	**Mixed Mongoloid, Aryan, and Aboriginal ancestry**	0.558 (144)	N/A	N/A	26.6° N
		≤−0.50	10.3	**6.72**	**15**	**258**	**Rural India** ^46^	**Not given**	0.624 (131)	N/A	N/A	16.4° N
		≤−0.50	10.3	**10.8**	**15**	**381**	**Urban India** ^40^	**Not given**	0.624 (131)	N/A	N/A	28.6° N
		≤−0.50	10.3	**9.6**	**15**	**326**	**Semi-urban South Africa** ^85^	**African, Indian, mixed**	0.666 (119)	N/A	372	29.9° S
		≤−0.50	10.3	**78.4**	**15**	**376**	**Urban China** ^48^	**Han (Chinese)**	0.738 (90)	518/494/531	N/A	23.1° N
		≤−0.50	10.3	**32.5**	**15**	**321**	**Urban Malaysia** ^49^	**Malay, Chinese, Indian and other**	0.789 (59)	N/A	465	3.3° N
		≤−0.50	10.3	**4.9**	**15**	**120**	**Iran** ^45^	**Not given**	0.774 (69)	N/A	436	32.4° N
		≤−0.50	10.3	**46.8**	**16**	**452**	**Rural China** ^39^	**Not given**	0.738 (90)	518/494/531	N/A	21.8° N
16–19	393	≤−0.50	12.7	**69.5**	**11–20**	**1249**	**Singapore** ^12^	**Chinese, Malay, Indian and others**	0.925 (5)	556/535/564	631	1.4° N
17	80	≤−0.50	15.0	**17.7**	**17**	**<1202**	**Australia** ^5^	**European Caucasian**	0.939 (2)	510/503/494	505	33.9° S
18–19	89	≤−0.50	16.9	**18.6**	**18–20**	**226**	**UK** ^41^	**White UK children**	0.909 (16)	500/497/493	N/A	54.8° N
[18.2 (±0.4)]	89	≤−0.25	18.0	**33.0**	**21.7 (±0.3)**	**112**	**Norway** ^55^	**Not given**	0.949 (1)	498/513/502	512	63.4° N

All results are based on cycloplegic autorefraction measurement, except for a few studies that used cycloplegic retinoscopy^38,40,46,50,53^, retinoscopy with tropicamide^43^ or cycloplegic subjective refraction^55^. Human Development Index (HDI) 2015^24^, mean score in Programme for International Student Assessment (PISA) 2015^82^, and average scale score for Trends in International Mathematics and Science Study (TIMSS) 2015^83^ for each country are listed (results for Norway in top row). N/A = Not participated (except from Malaysia which participated in PISA 2015, but did not meet the PISA response-rate standards). PISA results given for China are from the area Beijing-Shanghai-Jiangsu-Guangdong, and PISA results for UK are from Northern Ireland. The PISA 2015 OECD average in science/reading/mathematics = 493/493/490^82^, and TIMSS 2015 Scale Centerpoint for Mathematics 8th grade = 500^83^. Highest score is best. Latitude for each study region is given in the rightmost column (latitude for present study is 59.7–60.0° N).

While the prevalence of myopia is reported to have been rising around the world, a similar trend in Southeast Norway appears to be absent. Specifically, a 1971 study of 12–14-year-old Norwegian children in West Norway (latitude 60.4°) reported similar cycloplegic SERs to that found here (at latitude 59.7–60.0°), and similarly low myopia prevalence (SER ≤ −1.0) of 13.7% (Table 5)^53^. Interestingly, Fledelius reported stability in the myopia prevalence of Danish medical students over the period 1968–1998^54^. Moreover, the low rate of high myopia (0.5%; SER ≤ −6 D) observed here and the reported higher myopia prevalence in 21-year-olds in mid-Norway [myopia prevalence (SER ≤ −0.25) was 33% in the general population, latitude 63.4°]^55^ suggest that myopia onset is significantly delayed in Norwegians compared with East-Asians and some other Europe based populations^12,38,39,41,43,47^. The narrow range in refractive errors, higher prevalence of emmetropia with a hyperopic mean SER, coupled with a low prevalence of anisometropia and astigmatism lend support to this suggestion^56–58^. A further increase in myopia prevalence may be expected when the adolescents enter higher education^55^.

The education system in Norway is classified as high-performing^26^. The adolescents in this study spent >10 hours per day indoors doing near work including working on NED for >5 hours per day, which was comparable with the amount of time spent on NED reported in a study of sleep in 16–19-year-olds in West Norway (latitude 60.4°, n = 9,846)^59^. But, time spent on near work was not associated with myopia, as reported by others^60,61^, neither was total time spent on outdoor activities in the winter — the multivariate analyses showed that the association for other activities outdoors outweighed that of doing sports outdoors. There was, however, an association between myopia and less time spent outdoors in the summer holiday. Interestingly, the mean time spent outdoors in the winter [3.79 (±1.8) hours per day; data collection was February–March] was similar to that reported for East-Asian adolescents [*n* = 267; mean 3.79 (±1.9) hours per day]^12^ in Singapore, where there is no difference in daylight hours (12 hours per day) between seasons (Fig. 1). This parallel raises the question for Norwegian adolescents, as to why the potential negative consequences of limited daylight exposure during the long autumn-winter period, when there are fewer than 12 hours daylight per day (174 days, including 82 days in November–January with only 6–8 hours daylight per day), do not override the potential positive benefits of the long days during the shorter summer period (124 days with 15–19 hours daylight per day). Note that there is a ceiling effect to the benefits of long summer days, since several hours of the daylight are in the late evening or early hours of the morning when children and adolescents sleep^62,63^. Norwegian children most likely only have access to about 12 hours of the daylight available to them in the spring-summer period (Fig. 1), which is comparable to what the children in Singapore have access to every day of the year. Can the difference in myopia prevalence between Norwegian and for example Singaporean adolescents (12.7% versus 69.5%^12^) be down to the increased time Norwegian adolescents spend outdoors in the 8-week summer holiday only? Considering the effect on myopia progression reported from the outdoor activity clinical trials in East Asia^18^, it seems unlikely that this can be the case. This raises the further question in relation to whether exposure to daylight *per se* is the most important factor in the protective effect of outdoor activity [cf. Guggenheim *et al*.^64^]. Could the state of being well adapted to seasonal variations (circannual rhythms) be as important for coordinated eye growth as it is for general health^65^? Is this to a larger degree preserved in Norwegian adolescents, because of more outdoor time since early childhood?

Being outdoors is a part of the Norwegian culture and a major part of growing up. For example, children in Norwegian kindergartens are reported to spend 2 hours per day outdoors in the winter and at least 4 hours in the summer^66^. Furthermore, children are required to stay outdoors during school recess (three to five breaks that accumulates to at least 1 hour per day) all the way through primary school (6–12 years of age), and all year long^67^. Pre-adolescent children spend on average an additional 2 hours outdoors per day after school^68^. These exposure patterns are quite different from those of children attending East-Asian schools where recess time usually is spent indoors^13,17,18^. It has been suggested that 2 hours spent outdoors per day is needed to prevent onset of myopia^17^, with outdoor activities having a stronger protective effect in younger children (age 6 years vs. age 11–12 years)^19,69^. Our data for Norwegian adolescents represent further supportive evidence from a real-life experiment. Nonetheless, it is also possible that the early onset of myopia as observed in many East Asian populations may be driven by genetic predisposition more than by environmental factors^10,30^.

Sex differences in myopia prevalence have been reported previously^70–72^. As in past studies, females were found to have a higher prevalence of myopia than males. There was a significant correlation between AL and height in females, but not males, which may be related to the age of onset of the childhood growth spurt. Specifically, girls usually show an earlier growth spurt, starting approximately two years ahead of boys^73–75^. There is a parallel here with myopia onset for females, which has been reported to be two years ahead of males^54,75^. The implication of the earlier onset of myopia in females is that they have a higher risk for developing larger myopic errors and secondary ocular pathology — indeed, as reported for older age groups^76–78^.

Our study had several limitations. The sample size could have been larger with an even higher response rate, but this is comparable to other studies when considering the narrow age range (Table 5). The population studied may be biased in its representation, although we have shown our sample to be representative for the region of Norway from which it was drawn (see Supplementary Material). It was not representative in terms of sex, with a slightly higher number of females, but considering that more females were myopic this, if anything, might suggest that the true overall prevalence of myopia may be lower. The use of questionnaires for quantifying time outdoors is common in studies of refractive errors^11,69,79^, even though there are inherent limitations associated with such an instrument compared with objective measures, for example wearable light meters^80^. This includes analytical problems arising from the use of categorical responses to a continuous event. Nonetheless, the comparisons made above were limited to studies that also made use of questionnaires for quantifying time in the same way.

In summary, this cross-sectional study of adolescents in Southeast Norway revealed hyperopia to be the most common refractive error, with the prevalence of myopia being quite low, despite the few daylight hours in the autumn-winter period and high levels of indoor activity and near work. While the origin of refractive errors is likely multifactorial^56^, a dose-response relationship between daylight (outdoor exposure) and ocular axial elongation alone cannot explain the low prevalence in myopia, anisometropia and astigmatism in this population. Genetic and environmental risk factors may impact how refractive errors develop differently^81^, and our results may point to a lower genetic predisposition to myopia in this population. Alternatively, perhaps there is a particular combination of genetic predisposition, circannual adaptation, timing and pattern of exposure to myopia-generating environmental triggers that are effective in protecting the population at this latitude against myopia.

## Electronic supplementary material

Supplementary information about representativeness in the data.

## Data Availability

Supplementary data on the community profile and demographics, a more detailed summary of refractive errors, time spent on indoor and outdoor activities, and refractive errors of non-Norwegians (n = 46) are available at usn.figshare.com [10.23642/usn.6022790].
