# Supplementary material for: Prevalence and Possible Factors of Myopia in Norwegian Adolescents
Source: Sci Rep. 2018 Sep 7;8:13479. doi: 10.1038/s41598-018-31790-y (PMC6128933; doi:10.1038/s41598-018-31790-y)
Supplement: Supplementary file 1 — Supplementary information about representativeness in the data. [file 41598_2018_31790_MOESM1_ESM.pdf]

Supplementary Information

Prevalence and Possible Factors of Myopia in Norwegian adolescents

Lene A. Hagen, Jon V. B. Gjelle, Solveig Arnegard, Hilde R. Pedersen, Stuart J. Gilson, Rigmor C. Baraas

Table of Contents

Supplementary information about representativeness in the data…………………… 2

Table S 1. Distribution of age and sex in the schools' catchment area and total in Norway.. 2

Table S 2. Distribution of ethnicities in our sample, in the schools' catchment area, and total in Norway. 3

Table S 3. Level of education in the schools' catchment area and total in Norway. 4

Table S 4. Distribution of gross income in the schools' catchment area and total in Norway.. 5

Table S 5. Grade point average in our sample and total for the two schools enrolled in the study.. 6

References ……………………………………………………………………………..…7

Supplementary information about representativeness in the data

The catchment area of the two upper secondary schools included in this study consists of five municipalities with a total area of 2,906 m^2 (Flesberg, Kongsberg, Krødsherad, Modum, and Sigdal), and these are the only two upper secondary schools in the region. The Supplementary Tables S1–S4 show that the catchment area is a representative region of Norway in terms of distribution of age, sex, ethnicity and socio-demographic status as level of education and gross income. Supplementary Tables S2 and S5 show that our study sample is representative of the total population in the region and of the total population in Norway, with respect to distribution of ethnicity and grade point averages.

Table S 1. Distribution of age and sex in the schools' catchment area and total in Norway.

Distribution of age and sex given as number (*n*) and proportions (%) in the two schools' catchment area and total for the population in Norway in 2016.[1]

	The catchment area 2016 (*n* = 49,293)						Norway 2016 (*n* = 5,213,985)					
Age	Males		Females		Total		Males		Females		Total	
(yrs)	*n*	%	*n*	%	*n*	%	*n*	%	*n*	%	*n*	%
0–14	4,405	17.7	4,163	17.1	8,568	17.4	478,349	18.2	455,606	17.6	933,955	17.9
15–24	2,975	11.9	2,693	11.1	5,668	11.5	345,180	13.1	324,478	12.5	669,658	12.8
25–49	8,479	34.0	7,775	31.9	16,254	33.0	926,613	35.3	875,848	33.8	1,802,461	34.6
50–64	4,914	19.7	4,776	19.6	9,690	19.7	484,964	18.5	467,847	18.1	952,811	18.3
65–79	3,316	13.3	3,491	14.3	6,807	13.8	307,971	11.7	327,104	12.6	635,075	12.2
≥ 80	851	3.4	1,455	6.0	2,306	4.7	82,034	3.1	137,991	5.3	220,025	4.2

Table S 2. Distribution of ethnicities in our sample, in the schools' catchment area, and total in Norway.

Table showing distribution of ethnicities in our total sample (*n* = 439; age 16–19 yrs), for the two schools' catchment area population, and total for Norway.[2-4] Mixed ethnicity is defined as having parents of two different ethnicities.

Ethnicity	Our sample (*n* = 439)		Catchment area population 2016 (*n* = 49,293)[2-5]		Norway's population 2016 (*n* = 5,213,985)[4-6]	
	n	%	*n*	%	*n*	%
Norwegian	388	88.4	42,733	86.7	4,365,778	83.7
Total other	51	11.7	6,560	13.3	848,207	16.3
European *	11	2.5	3,699	7.5	430,671	8.3
Asian	24	5.5	1,710	3.5	265,721	5.1
African	6	1.4	745	1.5	114,304	2.2
South-American	4	0.9	286	0.6	24,256	0.5
North-American	0	0.0	107	0.2	11,072	0.2
Oceanian	0	0.0	13	0.0	2,183	0.0
Mixed	6	1.4	0	0.0	0	0.0

* European is defined here as originating from European countries other than Norway

Table S 3. Level of education in the schools' catchment area and total in Norway. Level of education[7] for 16 years and older males and females in the two schools' catchment area and total in Norway in 2015, given as numbers (*n*) and proportions (%).

Education	The catchment area October 2015 Total (*n* = 40,296)		Norway October 2015 Males (*n* = 2,117,010)		Females (*n* = 2,104,975)		Total (*n* = 4,221,985)	
	n	%	*n*	%	*n*	%	*n*	%
Below upper secondary education	11,049	27.4	570,495	26.9	557,697	26.5	1,128,192	26.7
Upper secondary education *	17,140	42.5	927,460	43.8	787,750	37.4	1,715,210	40.6
Higher education, short †	11,876	29.5	394,123	18.6	570,821	27.1	964,944	22.9
Higher education, long ‡			211,317	10.0	173,438	8.2	384,755	9.1
Unknown or no completed education §	231	0.6	13,615	0.6	15,269	0.7	28,884	0.7

* Includes intermediate level courses based on completed upper secondary level, but which are not accredited as tertiary education
† Comprises higher education up to 4 years in duration.
‡ Comprises higher education more than 4 years in duration.
§ For many immigrants Statistics Norway[7] has no information about their level of education.

Table S 4. Distribution of gross income in the schools' catchment area and total in Norway.

Gross income[8] for 17 years and older males and females in the catchment area and total in Norway, given as number (*n*) and proportions (%).

Gross income (NOK)	The catchment area 2015 (*n* = 39,532)		Norway 2015 (*n* = 4,150,990)	
	n	%	*n*	%
0–99,999	4,239	10.7	494,000	11.9
100,000–199,999	3,799	9.6	409,366	9.9
200,000 –299,999	6,617	16.7	657,469	15.8
300,000–399,999	6,856	17.3	701,412	16.9
400,000–499,999	5,850	14.8	640,716	15.4
≥500,000	12,171	30.8	1,248,027	30.1

Table S 5. Grade point average in our sample and total for the two schools enrolled in the study.

Grade point average (GPA) was calculated as the average of all grades at the end of lower secondary school for each student, with 60.0 as the best GPA possible. This table is showing the number (*n*) and the proportions (%) of GPA for our sample compared with all students who were in their 1st year of the two upper secondary schools in the same time period. Mean (SD) GPA was 40.8 (± 7.8) for our sample and 38.9 (± 8.5) for the 1st year population at the two schools. The majority of our participants finished lower secondary school in 2014 and 2015, and the average GPA for Norway was 40.4 in 2014[9] and 40.8 in 2015.[10]

GPA	Our sample (*n* = 455) *		1st year school population (*n* = 743)	
	n	%	*n*	%
NA	18	4.0	60	8.1
0.0 – 10.0	0	0.0	3	0.4
10.1 – 20.0	2	0.4	10	1.3
20.1 – 30.0	37	8.1	92	12.4
30.1 – 40.0	169	37.1	276	37.1
40.1 – 50.0	168	36.9	234	31.5
50.1 – 60.0	61	13.4	68	9.2

* Age: 16 – 19 yrs (*n* = 439), age: 20 – 24 yrs: (*n* = 16)

References

1. Statistics Norway. Tabell: 07459: Folkemengde, etter kjønn og ettårig alder. 1. januar (K) [Table: 07459: Population, by sex and age (1-year step). 1 January (M)]. 2016.
2. Regio AS. Innvandrerbefolkningen. Antall og andel. [Immigrants. Number and proportions.]. 2016.
3. Statistics Norway. Immigrants and Norwegian-born to immigrant parents, 1 January 2017. 2017.
4. Statistics Norway. Immigrants and Norwegian-born to immigrant parents. 2017.
5. Statistics Norway. Population and population changes. 2017.
6. Statistics Norway. Population and land area in urban settlements, 1 January 2016. 2017.
7. Statistics Norway. Population´s level of education, 1 October 2015. 2015.
8. Statistics Norway. Skattestatistikk for personer [Statistics of Personal Taxes]. 2017.
9. Statistics Norway. Marks, lower secondary school, 2014. 2014.
10. Statistics Norway. Marks, lower secondary school, 2015. 2015.
